# Feasibility, usability, and preliminary knowledge outcomes of a virtual-reality fire-safety training for undergraduate nursing students: a quasi-experimental study

**DOI:** 10.1186/s41077-026-00440-z

**Published:** 2026-05-08

**Authors:** Fatma Magdi Ibrahim, Ibrahim Al Faouri, Eman Abdelaziz Rashad Dabou

**Affiliations:** 1 RAK College of Nursing, RAK Medical and Health Sciences University, Ras Al Khaimah, UAE; 2 Faculty of Nursing, Mansoura University, Mansoura, Egypt; 3 Faculty of Nursing, Jordan University of Science and Technology, Irbid, Jordan; 4 Faculty of Nursing, Alexandria University, Alexendria, Egypt

**Keywords:** Virtual Reality, Disaster Preparedness, Fire Safety, Nursing Students, Simulation, System Usability Scale

## Abstract

**Background:**

In‑hospital fires are rare but high‑risk events, and nurses must be able to recognize hazards and initiate first‑response actions. This study compared a virtual‑reality (VR) fire‑safety training group with a time‑matched lecture group (covering the same learning objectives) and assessed system usability in the VR group among nursing students.

**Methods:**

We conducted a quasi-experimental study among undergraduate nursing students at RAK Medical and Health Sciences University, Ras Al Khaimah (RAK), United Arab Emirates. Two pre-existing timetable-based student groups, created by the university registrar during routine scheduling, were assigned as clusters to either the VR training group or a time-matched lecture group covering the same learning objectives. A researcher-developed 12-item fire-safety knowledge test (total score 0–12; 1 point per item) was administered before and after the session. Usability of the VR system was measured with the 10-item System Usability Scale (SUS; 0-100). Analyses included paired t-tests, chi-square tests, and analysis of covariance controlling for baseline knowledge (alpha = 0.05).

**Results:**

*N* = 130 students (VR group, *n* = 65; lecture group, *n* = 65) completed the study. Knowledge improved from 5.8 ± 1.2 to 9.3 ± 1.0 in the VR group (*p* < 0.001) and from 5.7 ± 1.3 to 7.8 ± 1.5 in the lecture group (*p* < 0.01). At post-test, 89% in the VR group vs. 62% in the lecture group achieved “good” knowledge (≥ 9/12; *p* = 0.002). ANCOVA indicated a significant between-group difference in post-test knowledge after adjustment for baseline (pre-test) knowledge, favoring the VR group. The VR system showed high usability (SUS = 84.6/100).

**Conclusion:**

Preparing nursing students to respond effectively to in-hospital fires is essential. In this pilot study, VR fire-safety training produced larger immediate knowledge gains than the time-matched lecture group and was rated highly usable, supporting its integration as a supplementary educational strategy.

**Supplementary Information:**

The online version contains supplementary material available at 10.1186/s41077-026-00440-z.

## Background

Although in-hospital fires are uncommon, they are high‑risk events because healthcare environments combine oxygen‑enriched areas, electrical equipment, and combustible materials. Fire incidents can lead to loss of life, injuries, service disruption, and property damage, and they often reveal gaps in fire-safety preparedness [1–5]. Nurses are frequently among the first professionals to respond on wards and must rapidly recognize hazards, initiate alarms, and implement first‑response actions; however, studies report insufficient fire‑safety knowledge and preparedness among healthcare professionals [6, 7].

Fire-safety education typically covers fundamental concepts (e.g., the fire triangle) and institutional protocols [8, 9], while broader fire-safety education literature emphasizes practical, evidence-based, behavior-focused approaches to hazard recognition and response [10]. However, conventional approaches such as lectures and written policies provide limited opportunity to rehearse time‑critical decision-making, evacuation actions, and extinguisher use under pressure. Active learning and simulation‑based education have been recommended for disaster preparedness because they support experiential practice and structured reflection [11–13].

Immersive virtual reality (VR) can provide a safe, interactive environment in which learners rehearse hazard recognition, alarm activation, evacuation decisions, and extinguisher use without exposing patients or staff to risk [14]. Evidence from health-professions education suggests that VR can improve engagement and, in some contexts, knowledge outcomes [15–18]. However, evidence specifically focused on undergraduate nursing students and fire-safety training remains limited; most published work involves perioperative teams or non-nursing populations [14]. In the United Arab Emirates (UAE), rapid urban development and high-rise infrastructure further strengthen the case for preparing nursing students for rare but high-risk in-hospital fire events [19].

Given this evidence gap, we developed and implemented a VR fire‑safety training session for undergraduate nursing students and evaluated its feasibility, usability, and preliminary knowledge outcomes.

This study aimed to (1) evaluate the feasibility of delivering a VR fire-safety training session (session completion, adverse symptoms such as dizziness or eyestrain, and technical issues); (2) assess the usability of the VR system using the System Usability Scale (SUS); and (3) compare fire-safety knowledge outcomes between the VR group and the lecture group using pre-/post-testing and a post-test analysis of covariance that controlled for baseline (pre-test) knowledge.

## Methods

### Design and reporting

We used a quasi-experimental, non-randomized cluster design. Before the study began, the university registrar had already created two timetable-based student groups for routine scheduling; these pre-existing groups were then assigned as clusters to either the VR group or the lecture group according to timetable feasibility and headset availability. Reporting follows the simulation-based research extensions to CONSORT and STROBE (CONSORT-SBR and STROBE-SBR) [20].

## Setting and participants

The study was conducted within a single undergraduate nursing program (Years 1–4) at RAK Medical and Health Sciences University in Ras Al Khaimah (RAK), United Arab Emirates. Eligible participants were undergraduate nursing students enrolled in one of the two selected timetable-based student groups, able to attend the scheduled VR group session or lecture group session, and willing to provide informed consent. Because allocation occurred at the student-group (cluster) level, the two groups were scheduled separately to minimize cross-exposure between interventions.

## Interventions

The VR intervention consisted of a single‑user immersive fire‑safety scenario delivered using Meta Quest 2 headsets with handheld controllers and a Unity‑based application developed by the research team. The scenario emphasized hazard recognition, first‑response actions using the RACE sequence (Rescue, Alarm, Confine, Extinguish/Evacuate) [21], fire‑extinguisher operation using the PASS technique (Pull, Aim, Squeeze, Sweep) [22], and safe egress strategies.

VR delivery: Students completed the VR scenario individually (one learner per headset; not a multiplayer environment) with a faculty facilitator present. Sessions were offered twice weekly over four weeks to accommodate scheduling and headset availability; each participant completed one VR session lasting approximately 30–45 min (including pre-briefing and debriefing). Each session included (a) an in-person pre-briefing and orientation (objectives, psychological safety, headset controls, boundaries, and motion-sickness mitigation), (b) faculty facilitation during scenario completion, and (c) an in-person debriefing immediately after the scenario, aligned with the Healthcare Simulation Standards of Best Practice of the International Nursing Association for Clinical Simulation and Learning (INACSL) [23]. Debriefing discussions were conducted in small groups to encourage peer reflection; learners could ask questions and share experiences during the facilitated debriefing and a structured question-and-answer discussion. Table 1 summarizes the alignment of learning objectives and instructional components across the VR and lecture groups.

**Table 1:** Intervention content and delivery (VR group vs. lecture group)

Component	VR group	Lecture group
Learning objectives/content	Hazard recognition; fire triangle; alarm activation; RACE actions; PASS extinguisher steps and extinguisher selection; evacuation routes and fire doors; unit‑level prevention measures.	Matched objectives/content to VR: hazard recognition; fire triangle; alarm activation; RACE actions; PASS steps and extinguisher selection; evacuation routes and fire doors; unit‑level prevention measures.
Delivery mode	Immersive interactive scenario in VR with facilitator support.	Instructor‑led didactic session using slides, demonstration images/videos, and guided discussion.
Prebriefing/orientation	In‑person orientation to headset controls, navigation, and motion‑sickness mitigation; objectives and psychological safety established.	In‑person orientation to session objectives and expectations; psychological safety and participation norms established.
Facilitation	Simulation‑trained faculty used a standardized script and checklist (Appendix 1) to ensure consistent cueing and support.	Instructor followed a standardized slide deck and discussion prompts to ensure consistent coverage and pacing.
Debriefing/reflection	Face‑to‑face debriefing immediately after scenario using a PEARLS‑type structure (reaction → analysis → summary).	Facilitated review and question-and-answer discussion at session end to reinforce key principles and address misconceptions.
Duration	Matched to lecture in total instructional time (including prebriefing and debriefing).	Matched to VR in total instructional time (including review and question-and-answer discussion).

Lecture delivery: The lecture group received an instructor-led session matched to the VR intervention in learning objectives and total instructional time (including an end-of-session review and question-and-answer discussion). The lecture covered fire-risk factors in healthcare, the fire triangle, prevention measures, alarm activation, RACE actions, PASS steps and extinguisher selection, evacuation routes and fire doors, and unit-level safety roles. Teaching methods included slides, instructor demonstration using images and videos of extinguisher components, and guided discussion with brief case vignettes. The lecture was delivered as a single session to each pre-existing timetable-based student group (approximately 30–35 students per session) and lasted approximately 30–45 min. No hands-on fire-extinguisher practice was included; learning activities were limited to instructor demonstration (images and videos), case vignettes, and guided discussion.

### Instructional design and theoretical underpinning

The VR intervention and its facilitated debriefing were designed using Kolb’s Experiential Learning Theory (concrete experience → reflective observation → abstract conceptualization → active experimentation) [24] and Cognitive Load Theory [25]. In practice, learners first completed an individual immersive scenario (concrete experience), then participated in a structured debriefing to reflect on actions and decisions (reflective observation), linked experiences to fire-safety principles and institutional protocols (abstract conceptualization) and discussed how they would apply RACE/PASS steps during future clinical placements (active experimentation). Design choices intended to manage extraneous cognitive load included a brief headset orientation, seated mode, and facilitator support during navigation.

### Outcomes and measures

Feasibility was assessed descriptively by recording session completion, adverse events (e.g., cybersickness), and technical issues during delivery of the VR sessions. Adverse symptoms (e.g., dizziness, nausea, eyestrain) and technical issues were recorded by facilitators on a structured session log (Appendix 1) as present/absent with brief descriptive notes. At the end of each VR session, participants were asked standardized questions about common VR-related symptoms, and facilitators also noted any symptoms observed during the session or spontaneously reported by participants.

Usability in the VR group was measured using the 10‑item SUS, scored from 0 to 100 according to the standard SUS procedure [26, 27]. The SUS is also widely used to evaluate perceived usability in educational technology contexts [28]. The SUS items were administered without modification (other than specifying “this VR training system” in place of “this system” for clarity) and were scored using the standard 0–100 procedure. Internal consistency in this sample was acceptable (Cronbach’s α = 0.75).

Preliminary learning outcomes were assessed using a 12-item multiple-choice fire-safety knowledge test (total score range 0–12; 1 point per item) administered pre- and post-intervention. The items were authored to align with the National Fire Protection Association (NFPA) 101 Life Safety Code and NFPA fire-safety education materials; no verbatim NFPA text or figures were reproduced [29]. Content covered extinguisher selection and PASS steps, alarm activation and RACE actions, evacuation routes and fire doors, and unit-level prevention measures. Each correct response received 1 point (incorrect/blank = 0). Internal consistency in the present study sample was high (Cronbach’s α = 0.86). The full 12-item test is provided in Appendix 2.

Paired t-tests assessed within-group pre- to post-intervention changes, and chi-square tests compared categorical variables between groups. For the primary between-group post-test comparison, we conducted an analysis of covariance (ANCOVA) with post-test knowledge score as the dependent variable, group (VR vs. lecture) as the fixed factor, and pre-test knowledge score as the covariate (alpha = 0.05). Analyses were performed using IBM SPSS Statistics version 29. For item-level post-test comparisons, we used Pearson chi-square tests (or Fisher’s exact test where expected counts were < 5).

### Sample size and analysis

**Fig. 1:**
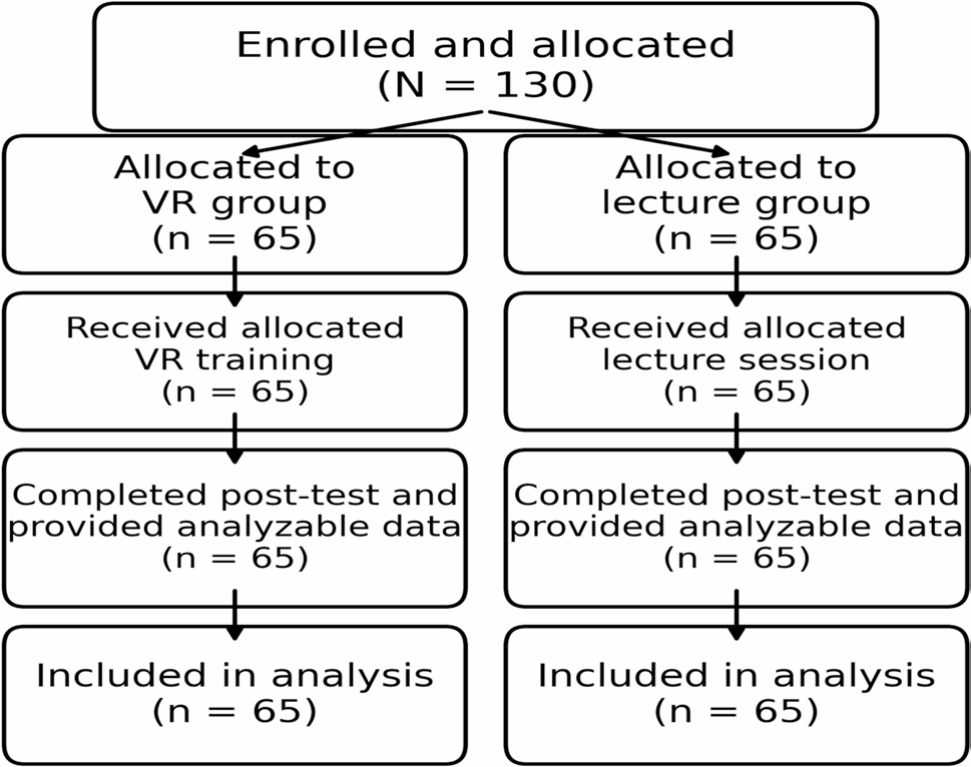
Participant flow diagram (no exclusions or withdrawals)

A prior power analysis (G*Power; ANCOVA, f = 0.25, alpha = 0.05, power = 0.80, one covariate) [30] indicated that ≥ 128 participants were required. A total of 130 undergraduate nursing students consented and were enrolled. No participants were excluded after enrolment and there were no withdrawals or losses to follow-up. Student‑group membership was determined before recruitment by the university registrar during routine scheduling (e.g., timetable and class-size constraints) rather than by the researchers. The two pre-existing timetabled student groups were allocated to the VR training group (*n* = 65) or the time-matched lecture group (*n* = 65) based on timetable feasibility and headset availability. All participants received the allocated session and completed the pre- and post-intervention knowledge assessments; the final sample analyzed included all 130 participants (VR group, *n* = 65; lecture group, *n* = 65) (Fig. 1). This sample size was considered pragmatic for assessing feasibility in this pilot study.

### Healthcare simulation standards of best practice^®^ (HSSOBP) compliance

During pre‑briefing, learners received written objectives, roles, and expectations. Psychological safety and confidentiality were emphasized. Orientation covered headset controls, navigation, and motion‑sickness mitigation.

Simulation sessions were led by simulation‑trained faculty using a standardized script. A facilitator checklist (Appendix 1) was used to support consistent delivery across cohorts.

Debriefing was conducted in person using a Promoting Excellence and Reflective Learning in Simulation (PEARLS)-type format (reaction → analysis → summary), a structured debriefing approach designed to support reflective learning in health care simulation [31]. The debriefing supported reflection on PASS protocols, alarm activation procedures, evacuation routes, and situational assessment. A debriefing guide/script is provided in Appendix 3.

Learning objectives were aligned with outcome measures (knowledge test; SUS). Fidelity elements (visual, conceptual, psychological, and procedural) were monitored using a facilitator checklist (Appendix 1) in line with simulation design guidance [23, 32]. Technical issues and adverse symptoms were logged. Core tasks included identifying ignition sources and hazards, selecting the appropriate extinguisher, performing PASS steps, activating alarms, choosing safe egress routes under time pressure, and verbally communicating actions.

Safety and hygiene procedures included brief acclimatization, guidance to reduce motion sickness, and headset disinfection and cover changes between users.

### Ethics

This study was conducted in accordance with the ethical principles of the Declaration of Helsinki. Approval was obtained from the RAK Medical and Health Sciences University Ethics Committee (Approval number: RAKMHSU-HEC-176-UG-N). Participant confidentiality was ensured using anonymized research identification codes, and all data were collected anonymously.

## Results

### Demographic Characteristics of Participants

The study comprised 130 nursing students, with 65 participants in each group. The average age was similar between groups, and most participants were in the 20–29 year age group (69.2% and 70.8% in the VR and lecture groups, respectively). Females constituted the majority in both groups (VR: 67.7%; lecture: 64.6%). Academic year distribution was relatively even across groups. Prior experience with VR technology was reported by 18.5% of participants in the VR group and 15.4% in the lecture group. No statistically significant between-group differences were detected for the measured baseline demographic variables (all *p* > 0.05) (Table 2).

**Table 2:** Socio-demographic Characteristics of Participants (*N* = 130)

	Variable	VR Group (*n* = 65)	Lecture Group (*n* = 65)	p-value
Age group	< 20 years	10 (15.4%)	9 (13.8%)	0.82
	20–29 years	45 (69.2%)	46 (70.8%)	
	30–39 years	7 (10.8%)	8 (12.3%)	
	40 + years	3 (4.6%)	2 (3.1%)	
Gender	Male	21 (32.3%)	23 (35.4%)	0.67
	Female	44 (67.7%)	42 (64.6%)	
Year of study	1st Year	17 (26.2%)	16 (24.6%)	0.91
	2nd Year	14 (21.5%)	13 (20.0%)	
	3rd Year	17 (26.2%)	18 (27.7%)	
	4th Year	17 (26.2%)	18 (27.7%)	
Previous VR experience	Yes	12 (18.5%)	10 (15.4%)	0.48
	No	53 (81.5%)	55 (84.6%)	

Chi-square test was used

*p* < 0.05 indicates statistical significance

Item-level post-test performance is presented in Table 3 and favored the VR group across all 12 questions (chi-square p-values shown). Total knowledge scores increased significantly from pre- to post-intervention in both groups (Table 4). In the VR group, mean knowledge increased from 5.8 (SD 1.2) to 9.3 (SD 1.0) (paired t-test, *p* < 0.001), whereas the lecture group increased from 5.7 (SD 1.3) to 7.8 (SD 1.5) (paired t-test, *p* < 0.01). At post-test, 89% of students in the VR group versus 62% in the lecture group achieved good knowledge (≥ 9/12; chi-square test, *p* = 0.002).

**Table 3:** Knowledge answers post-intervention between both groups

	VR group		Lecture group		
Question	Correct *n* (%)	Incorrect *n* (%)	Correct *n* (%)	Incorrect *n* (%)	*p*-value (χ²)
1. First step if fire is discovered	58 (89.2%)	7 (10.8%)	40 (61.5%)	25 (38.5%)	< 0.001
2. Fire hazard in healthcare	55 (84.6%)	10 (15.4%)	38 (58.5%)	27 (41.5%)	< 0.001
3. Safe fire evacuation route	60 (92.3%)	5 (7.7%)	42 (64.6%)	23 (35.4%)	< 0.001
4. Encountering smoke while evacuating	59 (90.8%)	6 (9.2%)	39 (60.0%)	26 (40.0%)	< 0.001
5. Extinguisher for electrical fires	57 (87.7%)	8 (12.3%)	41 (63.1%)	24 (36.9%)	0.001
6. Meaning of PASS	56 (86.2%)	9 (13.8%)	40 (61.5%)	25 (38.5%)	0.001
7. Fire prevention best practice	61 (93.8%)	4 (6.2%)	43 (66.2%)	22 (33.8%)	< 0.001
8. Damaged electrical equipment	54 (83.1%)	11 (16.9%)	38 (58.5%)	27 (41.5%)	0.002
9. Purpose of RACE	58 (89.2%)	7 (10.8%)	42 (64.6%)	23 (35.4%)	< 0.001
10. Action after activating alarm (RACE)	53 (81.5%)	12 (18.5%)	37 (56.9%)	28 (43.1%)	0.002
11. System triggered by smoke	60 (92.3%)	5 (7.7%)	41 (63.1%)	24 (36.9%)	< 0.001
12. Function of fire doors	55 (84.6%)	10 (15.4%)	39 (60.0%)	26 (40.0%)	0.002

n = numbers; % = percentages

**Table 4:** Comparison of Pre- and Post-Intervention Fire Safety Knowledge Scores

Group	Pre-Test Mean ± SD	Post-Test Mean ± SD	*p*-value
VR Group	5.8 ± 1.2	9.3 ± 1.0	< 0.001*
Lecture Group	5.7 ± 1.3	7.8 ± 1.5	< 0.01*

*Paired t-test within each group

*p* < 0.05 indicates statistical significance

Feasibility: All enrolled students completed the assigned training and post-test assessment (VR group, *n* = 65; lecture group, *n* = 65). During VR delivery, facilitators logged technical issues and adverse symptoms (e.g., cybersickness); no participant withdrew because of adverse symptoms, and no sessions were terminated due to technical problems. Facilitator logs documented two reports of mild transient cybersickness symptoms (one eyestrain, one dizziness) and one minor technical issue (temporary exit from the program); none required session termination (Appendix 1).

Usability: The VR system demonstrated high usability (overall SUS score = 84.6/100). Most participants agreed that the system was easy to use (mean = 4.5 ± 0.6) and that features were well integrated (mean = 4.4 ± 0.7) (Table 5).

**Table 5:** System Usability Scale (SUS) for VR Training (VR Group Only)

SUS Item	Mean ± SD
1. I think that I would like to use this VR training system frequently.	4.3 ± 0.8
2. I found the VR environment unnecessarily complex to navigate. (R)	2.1 ± 0.9
3. I thought the VR training system was easy to use.	4.5 ± 0.6
4. I think I would need the technical support of a person to use this system. (R)	2.2 ± 1.0
5. I found that the various features in this VR system were well integrated.	4.4 ± 0.7
6. I thought there was too much inconsistency in the VR interface. (R)	2.0 ± 0.8
7. I imagine most people would quickly learn to use this VR training system.	4.6 ± 0.5
8. I found the VR system cumbersome to operate. (R)	1.9 ± 0.9
9. I felt very confident using the VR training environment.	4.5 ± 0.6
10. I needed to learn many things before I could get going with this system. (R)	2.0 ± 0.7
Overall, SUS Score	**84.6**

(R) = Reverse-coded item

Adjusted between-group comparison: After controlling for baseline (pre-test) knowledge score in an ANCOVA, post-test knowledge remained significantly higher in the VR group than in the lecture group (Table 6; *p* < 0.001; partial η² = 0.262).

**Table 6:** ANCOVA of post-test knowledge score controlling for baseline (pre-test) knowledge

Model term	df	Statistic	*p*-value
**Group (VR vs. lecture)**	1, 127	F = 45.0; partial η² = 0.262	< 0.001

Estimated marginal (adjusted) post-test means (controlling for baseline knowledge): VR = 9.3; lecture = 7.8 (adjusted mean difference ≈ 1.5 points)

## Discussion

This quasi-experimental study examined the feasibility, usability, and preliminary knowledge outcomes of a VR fire-safety training program compared with a time-matched lecture in undergraduate nursing students. Delivery was feasible: all participants completed the assigned training and post-test, and no sessions were terminated because of adverse symptoms or technical problems. Usability was high (SUS = 84.6/100), and knowledge improved in both groups, with higher post-test scores in the VR group after controlling for baseline knowledge.

The stronger knowledge outcomes in VR are consistent with Kolb’s Experiential Learning Theory, in which concrete experience followed by structured reflection supports construction of actionable concepts [24]. Immersive rehearsal may help learners operationalize RACE/PASS decision points under time pressure while maintaining psychological safety through prebriefing, facilitation, and debriefing.

Our findings are consistent with broader evidence that immersive VR can enhance engagement and, in some contexts, knowledge outcomes in health-professions education when scenarios are well designed and supported by structured facilitation and debriefing [17, 18, 33, 34]. Recent immersive-learning theory also suggests that presence, affect, motivation, and cognitive load can shape how learners benefit from VR, which reinforces the importance of concise orientation and careful interface design [25, 35–37].

Resource considerations are important for implementation. A single educator can deliver a lecture to a large cohort at low marginal cost, whereas VR often requires headsets, technical support, and repeated sessions with smaller numbers of learners. In our setting, offering multiple VR time slots and facilitating individual headset use increased staffing and scheduling demands. However, once developed, VR scenarios can be reused, standardized, and scaled across cohorts with consistent content delivery and without physical consumables, and may reduce reliance on physical simulation space or equipment. Future work should include formal cost and cost-effectiveness analyses alongside educational outcomes to inform curriculum decisions.

High usability ratings suggest that the chosen hardware, software, and facilitation approach supported learners' interaction with the system. Brief orientation, low-stakes acclimatization, and structured debriefing may help reduce the initial learning curve for learners who are new to VR and support reflective learning after immersive simulation, consistent with prior usability, e-learning, and simulation-debriefing literature [28, 31, 38].

Strengths of this pilot study include the use of a time-matched comparison group, alignment with the Healthcare Simulation Standards of Best Practice^®^, and the use of both an objective knowledge test and a standardized usability instrument. To support replicability, the facilitator fidelity checklist is included (Appendix 1), together with the fire-safety knowledge test (Appendix 2) and the debriefing guide/script (Appendix 3).

Limitations include single-site delivery, non-random allocation of two pre-existing timetable-based student groups (cluster assignment), and short-term outcome measurement. Because these groups were created by the university registrar during routine scheduling and were assigned according to timetable feasibility (rather than by the investigators), selection bias and residual confounding cannot be excluded. We did not assess psychomotor performance (e.g., extinguisher technique), team behaviors, cognitive load, or longer-term retention of fire-safety knowledge. Because the comparator was a time-matched lecture, the observed differences may reflect the combined effects of immersive exposure, facilitation, and debriefing rather than VR alone. With only two clusters available, analyses were conducted at the individual level and do not account for intra-cluster correlation; findings should therefore be interpreted as preliminary. Future multi-site studies should compare VR with conventional simulation modalities, include follow-up assessments, and incorporate performance-based outcomes while accounting for clustering.

## Conclusion

Compared with a time-matched lecture covering the same learning objectives, VR fire-safety training was feasible to deliver, was rated highly usable, and produced larger immediate knowledge gains among undergraduate nursing students. VR may be a useful supplement to existing instructional methods for rehearsing RACE and PASS actions and evacuation decisions before clinical placement. Larger multi-site studies should test longer-term knowledge and performance outcomes and compare VR directly with other simulation modalities.

## Supplementary Information

Below is the link to the electronic supplementary material.

Supplementary Material 1.

## Data Availability

Materials will be available from the corresponding author on reasonable request.

## Appendix 1. Fidelity checklist used to monitor delivery and scenario integrity

**Table:**

Checklist item	Completed (Yes/No) / Notes
Prebriefing delivered (objectives, roles, confidentiality, psychological safety).	Yes
Learner orientation completed (headset controls, boundaries, seated mode, safety straps).	Yes
Motion‑sickness mitigation reviewed (stop signal, optional breaks, seated use).	Yes
Scenario started correctly; audio and visuals functioning.	Yes
Core hazard cues visible/available for identification.	Yes
PASS extinguisher sequence prompted/available in scenario.	Yes
RACE response sequence prompted/available in scenario.	Yes
Egress route decision point presented (e.g., smoke/blocked path cues).	Yes
Facilitator followed standardized script; minimal coaching beyond protocol.	Yes
Debriefing completed (PEARLS structure) and key learning objectives reviewed.	Yes
Technical issues documented (if any).	Yes; one student accidentally exited the VR application, but progress was saved and the student re-entered and completed the scenario.
Adverse symptoms documented (if any).	Yes; two students reported mild discomfort while wearing the headset (one eyestrain, one dizziness), but both completed the session without withdrawing.

## Appendix 2. Fire-safety knowledge test (12 items)

Instructions: Select the one best answer for each question. Each correct answer = 1 point (total score range 0–12).

1.  The fire triangle is made up of:

(A)  Heat, water, and oxygen
(B)  Fuel, heat, and oxygen
(C)  Smoke, fuel, and water
(D)  Heat, carbon dioxide, and fuel

2.  In the RACE sequence, the letter “A” stands for:

(A)  Alert
(B)  Alarm
(C)  Assist
(D)  Assess

3.  Which action should generally be performed first when a fire is discovered in a patient care area?

(A) Attempt to extinguish the fire immediately
(B) Rescue people in immediate danger
(C) Open all doors to improve visibility
(D) Turn off the fire alarm to reduce panic

4.  In the PASS technique for operating a fire extinguisher, “S” stands for:

(A)  Stop
(B) Sweep
(C) Squeeze
(D)  Secure

5.  The most appropriate first step if you notice smoke coming from an electrical device is to:

(A)  Pour water on the device
(B)  Activate the alarm and follow RACE actions
(C)  Unplug the device and continue patient care
(D)  Cover the device with a blanket

6.  A fire door is primarily used to:

(A)  Keep the unit quiet
(B)  Confine smoke and fire and slow spread
(C)  Improve ventilation during an emergency
(D)  Provide an exit only for staff

7.  Which extinguisher type is generally appropriate for ordinary combustibles such as paper and bedding?

(A)  Class A
(B)  Class B
(C)  Class C
(D)  Class D

8.  When using a fire extinguisher, the nozzle should generally be aimed:

(A)  At the flames at eye level
(B)  At the base of the fire
(C)  At the ceiling to disperse heat
(D)  At the nearest window

9.  If the fire is growing or smoke is heavy, the best action is to:

(A)  Continue trying to extinguish it alone
(B)  Evacuate/relocate as per protocol and close doors behind you
(C)  Silence the alarm to focus on the fire
(D)  Wait for instructions before doing anything

10.  Which statement best describes why oxygen therapy areas are higher risk during a fire?

(A)  Oxygen reduces combustion
(B)  Oxygen-enriched environments can accelerate fire growth
(C)  Oxygen makes smoke less toxic
(D)  Oxygen prevents ignition sources from working

11.  In a hospital, evacuation is usually best described as:

(A)  Always a full building evacuation immediately
(B)  Typically a staged approach (e.g., horizontal then vertical) based on risk and policy
(C)  Only moving staff, not patients
(D)  Optional if alarms are loud

12.  Which information is most important to communicate when activating an alarm/calling for help?

(A)  Your academic year
(B)  The fire location and what is burning (if known)
(C)  The number of extinguishers on the unit
(D)  The time the shift started

Answer key (for research replication): 1B, 2B, 3B, 4 C, 5B, 6B, 7 A, 8B, 9B, 10B, 11B, 12B.

## Appendix 3. Debriefing guide/script (PEARLS-type)

Facilitator note: Use a supportive tone. Maintain psychological safety. Keep the debriefing focused on the learning objectives.

1) Reaction (2–3 min).

- “How did that feel?”
-  “What stood out to you most during the scenario?”

2) Analysis (8–12 min).

Hazard recognition.

-  “What cues suggested a fire risk or an active fire?”
-  “What hazards did you identify (e.g., oxygen sources, electrical equipment, combustibles)?”

RACE actions.

-  “Walk me through what you did first after noticing the fire/smoke.”
-  “How did you prioritize Rescue, Alarm, Confine, and Extinguish/Evacuate?”
-  “What would you do differently if the patient was non-ambulatory?”

PASS and extinguisher use.

-  “When is it appropriate to use an extinguisher versus evacuate/relocate?”
-  “Describe PASS. Where should you aim the nozzle?”
-  “What extinguisher type would you choose for an electrical fire?”

Evacuation and containment.

-  “What role do fire doors and closed doors play?”
-  “How would you follow unit policy for horizontal and/or vertical evacuation?”

Communication and teamwork.

-  “Who would you notify and what key information would you communicate?”
-  “What role does delegation play during a fire response?”

3) Summary (2–3 min).

-  “What are your top three take-home points from today?”
-  “In your next clinical placement, how will you apply RACE/PASS and evacuation principles if a fire occurs?”
-  “What resources (unit maps, fire exits, extinguisher locations) will you check in advance?”

## Abbreviations

ANCOVA: Analysis of Covariance
CONSORT‑SBR: CONSORT extension for simulation‑based research
HSSOBP: Healthcare Simulation Standards of Best Practice
INACSL: International Nursing Association for Clinical Simulation and Learning
NFPA: National Fire Protection Association
PASS: Pull, Aim, Squeeze, Sweep
PEARLS: Promoting Excellence and Reflective Learning in Simulation
RACE: Rescue, Alarm, Confine, Extinguish/Evacuate
STROBE‑SBR: STROBE extension for simulation‑based research
SUS: System Usability Scale
UAE: United Arab Emirates
VR: Virtual Reality
