# Supplementary figures and images for: Feasibility, usability, and preliminary knowledge outcomes of a virtual-reality fire-safety training for undergraduate nursing students: a quasi-experimental study
Source: Adv Simul (Lond). 2026 May 8;11:47. doi: 10.1186/s41077-026-00440-z (PMC13326246; doi:10.1186/s41077-026-00440-z)

**Figure 1: The virtual reality fire safety training setup used in this study.**


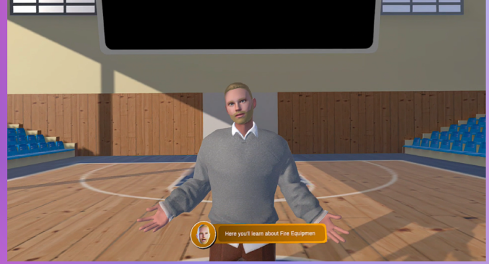

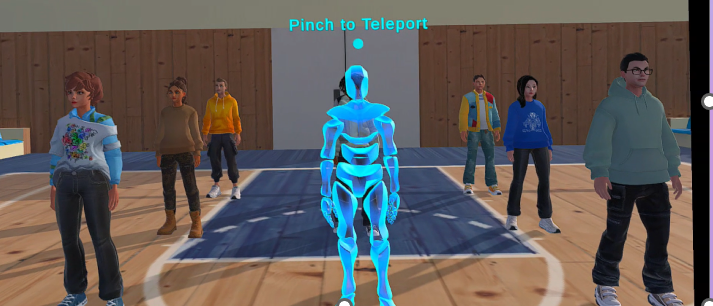

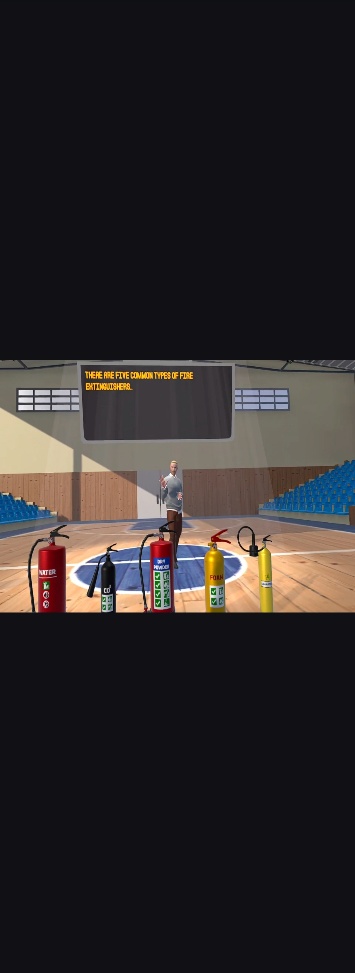

Supplement: Supplementary file 1 — Supplementary Material 1. [file 41077_2026_440_MOESM1_ESM.docx]
